# Supplementary material for: The difference in expression of long noncoding RNAs in rat semen induced by high-fat diet was associated with metabolic pathways
Source: PeerJ. 2017 Jul 25;5:e3518. doi: 10.7717/peerj.3518 (PMC5530988; doi:10.7717/peerj.3518)
Supplement: Data S1 [file peerj-05-3518-s001.doc]

**Effect of DIO rats on GLU, HDL, LDL, TG and body weight.**

| **Machine number** | **GLU mmoI/L** | **HDL-C mmoI/L** | **LDL-C mmoI/L** | **TG mmoI/L** | **Body Weight (g)** |
| --- | --- | --- | --- | --- | --- |
| **Normal** |  |  |  |  |  |
| G423 | 1.68 | 0.38 | 0.14 | 0.38 | 580 |
| G424 | 6.53 | 0.44 | 0.12 | 0.34 | 610 |
| G426 | 9.56 | 0.56 | 0.06 | 0.4 | 630 |
| G427 | 8.79 | 0.61 | 0.08 | 0.22 | 560 |
| G428 | 6.23 | 0.47 | 0.31 | 0.26 | 605 |
| G429 | 7.92 | 0.47 | 0.1 | 0.32 | 600 |
| G430 | 7.02 | 0.36 | 0.09 | 0.3 | 620 |
| mean | 7.22 | 0.47 | 0.13 | 0.31 | 600.71 |
| **HFD** |  |  |  |  |  |
| G431 | 8.92 | 0.62 | 0.17 | 0.66 | 690 |
| G432 | 8.37 | 0.55 | 0.11 | 0.17 | 705 |
| G433 | 8.72 | 0.53 | 0.09 | 0.43 | 780 |
| G434 | 9.16 | 0.5 | 0.1 | 0.32 | 705 |
| G435 | 8.52 | 0.52 | 0.09 | 0.28 | 720 |
| G436 | 7.98 | 0.42 | 0.05 | 0.33 | 710 |
| G437 | 6.93 | 0.44 | 0.09 | 0.37 | 745 |
| mean | 8.37 | 0.51 | 0.1 | 0.37 | 722.14 |
